# Artificial intelligence for automated detection of interictal epileptiform discharges: methodological progress, clinical evaluation, and neurodevelopmental context

**DOI:** 10.3389/fcell.2026.1941616

**Published:** 2026-09-08

**Authors:** Xinnan Ma, Pinchun Wang, Jingyou Ma, Yeting Lu, Shengjie Pan, Xiaowei Hu

**Affiliations:** Department of Neurology, The First Affiliated Hospital of Soochow University, Suzhou, China

**Keywords:** clinical validation, deep learning, electroencephalography, human–AI collaboration, interictal epileptiform discharges, neurodevelopment

## Abstract

Interictal epileptiform discharges (IEDs) are established electroencephalographic biomarkers that support epilepsy diagnosis and characterization, but their visual identification is time-consuming and subject to inter-rater variability. This review critically examines automated IED analysis from rule-based systems to contemporary deep learning, with particular attention to task definition, validation design, and clinical workflow. We use the term “accuracy paradox” as an author-defined descriptive label—not as an established field-wide concept—for the mismatch between high performance on presegmented epochs or recording-level classification and the temporal and spatial precision required for event-level interpretation. Accuracy, area under the curve, F1 score, concordance, and false positives per hour quantify different aspects of performance and should not be treated as interchangeable. Clinical evaluation should therefore specify the target event, temporal and spatial matching rules, recording duration, annotated event count, validation level, and intended use, while reporting sensitivity, precision, F1 score, false-positive burden, and workflow outcomes as appropriate. We also discuss neurodevelopmental and cellular mechanisms that may influence network excitability, including neuroinflammation, complement-associated synaptic remodeling, and chloride homeostasis. Available evidence supports biological plausibility but does not establish that AI-derived IED morphology can reveal molecular states in individual patients. We propose a translational framework based on independent multicenter validation, context-specific operating thresholds, robust evaluation of explanations, and human-in-the-loop triage. Prospective studies integrating scalp or intracranial EEG with spatially matched surgical tissue and single-cell or spatial transcriptomic profiling could test macro-to-molecular hypotheses, but these applications remain investigational.

## 1 Introduction

Epilepsy is a common chronic neurological disorder characterized by a persistent predisposition to epileptic seizures and abnormal network excitability. Although clinical history remains central to diagnosis, electroencephalography (EEG) provides essential physiological evidence for classification and management (McDougall et al., 2023).

Interictal epileptiform discharges (IEDs), including spikes, sharp waves, and spike–slow-wave complexes, provide important supporting evidence when an overt seizure is not captured during routine EEG (Kural et al., 2020). Their spatial distribution may overlap with elements of the seizure-onset network and can reflect abnormal synchronization within local and distributed circuits (Smith et al., 2022; Henin et al., 2021; Fouad et al., 2022). Systematic detection of these nonrandom electrophysiological events is therefore relevant to epilepsy assessment, while their interpretation must remain anchored to clinical context (McDougall et al., 2023).

IEDs may also carry prognostic and functional information. In adults with epilepsy, their presence on routine EEG has been associated with a higher risk of seizure recurrence (Lemoine et al., 2025). Transient interictal activity can interfere with ongoing cognitive processing, including memory and language, depending on its timing and anatomical distribution (Silva et al., 2023). In children, the relative cognitive burden of IEDs and antiseizure medications (ASMs) has also been investigated (Warsi et al., 2023). IEDs are not specific to epilepsy, however; they may occur in people without clinical seizures and have been reported in neurodegenerative disorders, where their significance remains an active area of study (Lemus and Sarkis, 2023).

Visual identification by trained clinical neurophysiologists remains the reference standard for IED interpretation, but it is labor-intensive and subject to intra- and inter-rater variability (Kural et al., 2020; Lodder and van Putten, 2014; Sun et al., 2025). The increasing use of video-EEG and continuous EEG has further expanded the volume of data requiring review (Lemoine et al., 2025; Montenegro et al., 2025; Liu et al., 2023). Longer recordings also increase the likelihood of incidental or equivocal findings. In critically ill patients, rhythmic or periodic patterns on the ictal–interictal continuum may create treatment uncertainty and should not be conflated with isolated IEDs. Automated output must therefore be interpreted together with montage, artifacts, neuroimaging, medication exposure, and the clinical history (Montenegro et al., 2025).

**FIGURE 1:**
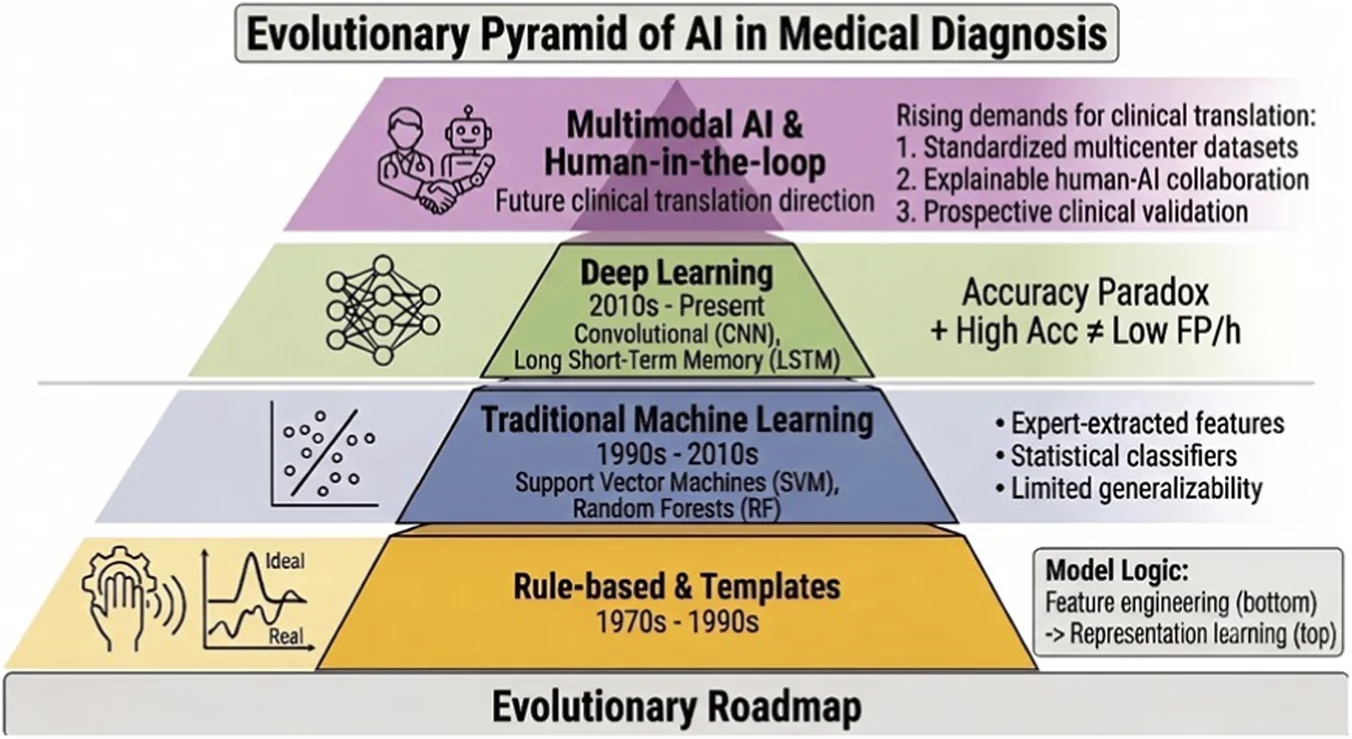
Methodological evolution and translational requirements for automated IED analysis.

These practical constraints motivate automated IED analysis, but reported performance can be difficult to interpret because studies often evaluate different targets. In this review, we use “accuracy paradox” as an author-defined shorthand for a metric–task mismatch: high accuracy on preselected epochs or recording-level labels may coexist with inadequate localization of individual discharges or an unmanageable candidate burden. The term is not intended to imply an established consensus construct. We organize the literature around three translational barriers: heterogeneous task definitions and metrics, limited external validation, and uncertain clinical interpretability. We then outline a human–AI workflow in which algorithms prioritize candidate events while clinicians retain responsibility for interpretation and diagnosis (Figure 1).

Scalp EEG represents the summed activity of neuronal populations and is shaped by network geometry, synaptic currents, developmental stage, behavioral state, medication, and recording conditions. It can therefore provide indirect information about network physiology, but it is not a direct molecular readout. The biological sections below are included to frame plausible determinants of IED variability and to identify testable hypotheses; they should not be interpreted as evidence that a detector can infer a specific cellular mechanism from waveform morphology alone.

The pyramid summarizes the transition from rule-based and feature-engineered methods to deep learning and human-in-the-loop decision support. The “accuracy paradox” is used here only as an author-defined label for the mismatch between strong performance on a coarse classification task and inadequate performance for the intended clinical task. Translation requires explicit task definitions, patient-independent data partitioning, independent external validation, event-level and recording-level evaluation, context-specific false-positive assessment, and measurement of workflow outcomes rather than reliance on a single summary metric.

## 2 Cellular origins and molecular determinants of IEDs

At the network level, IEDs are transient, spatially organized electrophysiological events generated by abnormal synchronization and propagation across neuronal populations (Smith et al., 2022; Henin et al., 2021; Fouad et al., 2022). Automated systems attempt to recognize the resulting waveform and field distribution. Successful detection does not, by itself, quantify the excitatory–inhibitory balance or identify the cellular process that generated a particular discharge.

### 2.1 Glial microenvironment and neuroinflammatory modulation

Glial cells and inflammatory signaling can influence neuronal excitability. Activated microglia, altered astrocytic function, and mediators such as interleukin-1β and tumor necrosis factor-α can modify synaptic transmission, receptor trafficking, and ionic homeostasis through several signaling pathways (Vezzani et al., 2019). These mechanisms provide a biologically plausible source of state-dependent network variability. Direct evidence that a defined inflammatory state produces a specific, reproducible IED morphology that can be decoded by AI is nevertheless limited. Neuroinflammation should therefore be presented as mechanistic context and a hypothesis for future paired studies, not as an established explanation for waveform-level model output.

### 2.2 IEDs as markers and potential modifiers of network plasticity

IEDs can transiently disrupt physiological hippocampal–cortical communication and interfere with memory-related activity, supporting the possibility that a high IED burden contributes to impaired cognition or altered plasticity in some settings (Montenegro et al., 2025). At the same time, IEDs may be consequences or markers of an already abnormal network rather than primary drivers of remodeling. Their causal contribution is likely to depend on developmental stage, discharge frequency, anatomical distribution, sleep state, and the underlying epilepsy syndrome. Accordingly, longitudinal IED burden is best interpreted as a candidate biomarker and potential modifier, not as a universally causal mechanism.

### 2.3 Developmental synaptic pruning and structural remodeling

Genetic and developmental processes can shape network excitability and EEG phenotype. SCN1A-related Dravet syndrome illustrates how an ion-channel disorder may be associated with age-dependent electroclinical features, but it is not representative of all genetic epilepsies (Catterall, 2014). Considerable overlap exists among EEG phenotypes across genetic disorders; consequently, EEG-based genotype prediction remains exploratory and is not a substitute for molecular testing (Abbasi and Goldenholz, 2019). Developmental synapse elimination is also regulated by neuron–glia signaling, including complement-associated microglial pathways (Wilton et al., 2019). Experimental evidence links disruption of these pathways to altered synaptic architecture and network excitability, but direct validation in human IED phenotypes remains limited. These observations support hypothesis generation rather than deterministic molecular interpretation of EEG.

### 2.4 Bridging cellular mechanisms to algorithmic design

Taken together, developmental stage, vigilance state, medication exposure, network anatomy, and cellular environment may contribute to IED heterogeneity. These considerations motivate age-aware and state-aware model development, but a biologically informed design should not be assumed to generalize better without empirical testing. Robustness must be demonstrated across sleep and wakefulness, age groups, recording systems, institutions, and clinically relevant artifacts (Smith et al., 2022; Henin et al., 2021; Fouad et al., 2022).

### 2.5 Developmental chloride homeostasis and IED generation

The effect of γ-aminobutyric acid (GABA) depends partly on the neuronal chloride gradient regulated by cation–chloride cotransporters, including NKCC1 and KCC2 (Kaila et al., 2014). Developmental trajectories of these transporters vary by brain region, cell type, and developmental stage; GABAergic signaling therefore cannot be reduced to a universal switch from “excitatory in early development” to “inhibitory after maturation” (Kaila et al., 2014; Ben-Ari et al., 2012). In some experimental models and resected human temporal-lobe tissue, impaired chloride extrusion or altered KCC2/NKCC1 function has been associated with depolarizing GABA responses and hyperexcitability (Huberfeld et al., 2007). These findings do not establish a uniform chloride abnormality across epilepsy syndromes. For automated analysis, the practical implication is that age, brain region, sleep state, and syndrome should be modeled or stratified rather than treated as interchangeable sources of EEG data.

**FIGURE 2:**
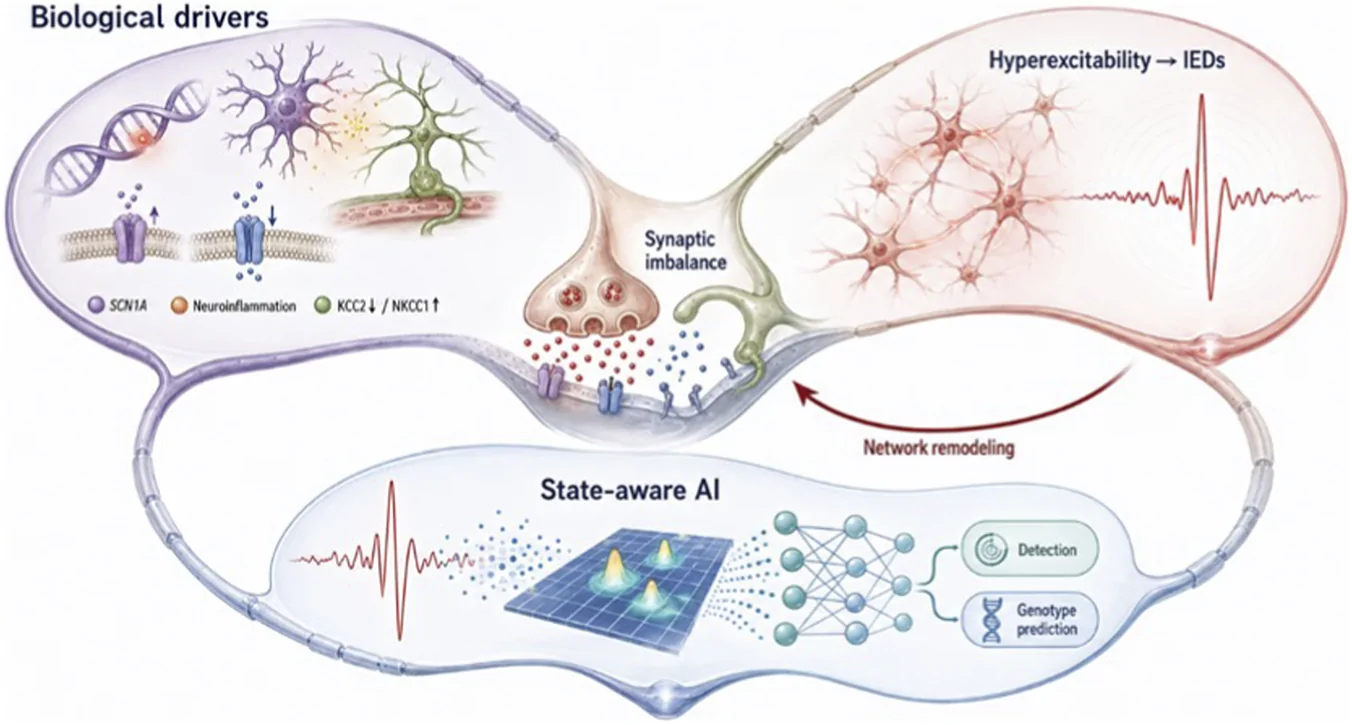
Conceptual relationships among biological determinants, network excitability, IEDs, and state-aware EEG analysis.

Figure 2 presents these relationships as a conceptual framework rather than a validated causal chain. Genetic variation, inflammatory signaling, synaptic remodeling, and chloride regulation may influence network excitability and IED expression. AI can quantify EEG features and support detection or phenotyping, whereas any inference about genotype, cell state, or molecular pathway requires independent biological validation.

Genetic variation, neuroinflammatory signaling, synaptic remodeling, and altered chloride regulation are examples of processes that may affect neuronal synchronization and IED expression. The arrows indicate plausible relationships supported to different degrees by experimental and clinical evidence; they do not imply that every pathway is present in every patient or that IEDs invariably drive network remodeling. State-aware AI may improve detection and quantitative EEG phenotyping, but EEG-based genotype or molecular prediction remains investigational and requires external biological validation.

## 3 Foundations and early approaches to automated IED detection

Early automated IED detectors attempted to reproduce visual criteria through waveform templates, thresholds, and hand-crafted morphological rules. These methods were transparent and computationally efficient, but normal variants, muscle and eye-movement artifacts, and the heterogeneity of true IEDs frequently produced false detections. Performance also depended strongly on the population, montage, filtering, and recording environment (da Silva Lourenço et al., 2021).

One line of work operationalized criteria proposed by the International Federation of Clinical Neurophysiology (IFCN), including sharpness, duration, amplitude, background disruption, and an associated slow wave (Nascimento et al., 2023). Quantifying these features helped translate expert descriptors into measurable variables. However, criterion-based systems remain vulnerable to low-amplitude discharges, atypical morphology, and artifacts that satisfy only part of the criteria.

Probabilistic approaches subsequently represented the likelihood that a candidate event was epileptiform rather than forcing a binary decision (Abdi-Sargezeh et al., 2021). This formulation is useful because expert labels themselves may be uncertain. The estimated probability nevertheless depends on the training labels, feature representation, and calibration of the model; it should not be interpreted as an objective biological probability without validation.

These early methods remain conceptually important, but their clinical evaluation was often incomplete. Many studies reported segment-level sensitivity or accuracy without recording duration, event counts, precision, or false-positive burden. False positives per hour (FP/h) are useful in long recordings, but they are not the only relevant outcome and must be interpreted in the intended workflow. In addition, testing on different patients from the same center is internal patient-level validation, not external validation. External validation requires an independent institution or dataset and should ideally include different acquisition hardware or protocols.

## 4 Traditional machine learning approaches

Traditional machine learning reframed IED analysis as supervised classification using features from the time, frequency, wavelet, or network domains. Support vector machines, random forests, and boosting methods can separate candidate transients from background more flexibly than fixed templates, while retaining some visibility into the engineered features (Falach et al., 2024).

Several intracranial EEG studies combined IEDs with high-frequency oscillations and other quantitative features to classify epileptogenic tissue or estimate an epileptic zone (Klimes et al., 2019). Such work is relevant to presurgical localization, but its outcome is not the same as detecting individual IEDs. Zone-classification accuracy should therefore not be used as direct evidence of event-level IED detection performance.

Other studies integrated graph-theoretical features or shallow neural networks to characterize interictal networks and improve focus localization (Balaji and Parhi, 2025). Gradient-boosting approaches have also reported high cross-validated sensitivity and accuracy for labeled iEEG segments (Falach et al., 2024). These results demonstrate the value of multidimensional features, but cross-subject testing within a source dataset should not be described as external or cross-center validation.

Traditional machine learning can also model background abnormalities that are not reducible to visually obvious spikes (Thangavel et al., 2022). This broader capability may support recording-level screening, but the target label—individual IED, abnormal epoch, epilepsy diagnosis, or epileptogenic zone—must be stated explicitly because each target requires different annotations and metrics.

Efficient machine-learning classifiers may be useful as a first-stage filter that removes obvious background and sends a smaller candidate set to a more computationally intensive model or to an expert (Bagheri et al., 2019). The benefit of such a cascade should be evaluated through candidate burden, sensitivity after filtering, and review time rather than computational speed alone.

Overall, traditional machine learning improved flexibility and artifact discrimination compared with fixed rules, but many studies relied on small, single-center datasets and dataset-specific features. Their contribution should be interpreted in relation to the task evaluated, the unit of analysis, and the validation design. Lack of FP/h reporting does not invalidate a study, but it limits conclusions about deployment in long-term monitoring.

## 5 Advances in deep learning for automated IED detection

### 5.1 From feature engineering to automated feature learning

Deep learning uses multilayer neural networks to learn representations from raw or minimally processed EEG. Convolutional and recurrent architectures can reduce reliance on manually selected features, but their performance remains dependent on sampling strategy, annotation quality, class balance, preprocessing, and the relationship between training and test data.

Convolutional neural networks (CNNs), long short-term memory networks (LSTMs), and related architectures have been applied to IED candidate classification, recording-level interpretation, spatial classification, and artifact reduction. These are distinct tasks. A higher score for one task does not establish superiority for another, and broad claims of generalization require independent testing.

### 5.2 Metric–task mismatch: Window or recording classification versus event localization

**FIGURE 3:**
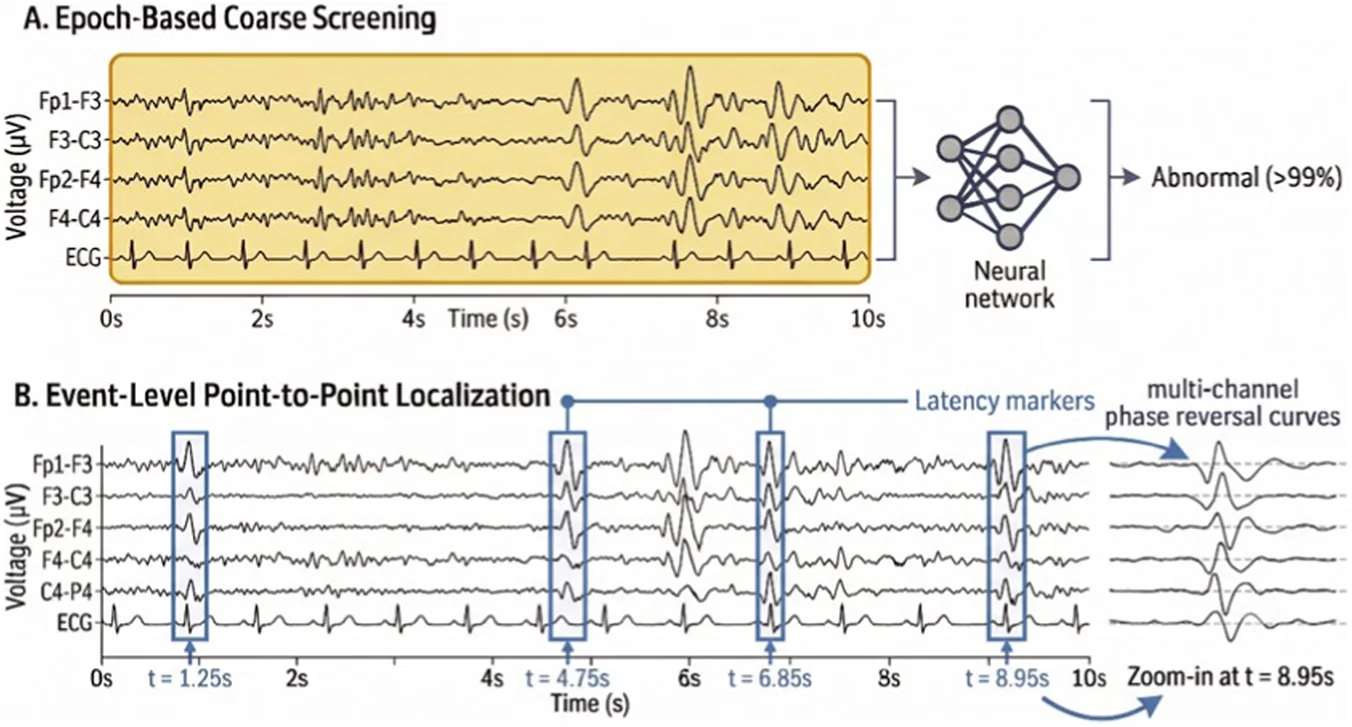
Schematic distinction between coarse window classification and event-level localization with prespecified matching rules. **(A)** Window-level classification assigns a label or probability to a fixed-duration EEG segment. It can be useful for screening but does not necessarily identify the onset, peak, offset, or spatial field of an individual IED. **(B)** Event-level localization marks candidate discharges and may additionally evaluate channel or field distribution. The illustrated timestamps are schematic rather than a recommended scoring tolerance. A valid benchmark must state what is annotated, how temporal overlap or peak latency is matched, whether spatial agreement is required, and how multiple adjacent events are handled.

Deep-learning studies often report strong results, but the unit of evaluation varies substantially. Some models classify a presegmented window, some determine whether a full EEG contains IEDs, and others attempt to mark individual discharges. Figure 3 schematically illustrates the distinction between window-level classification and event-level localization. These questions are clinically related but not equivalent. The central issue is therefore not that one paradigm is intrinsically valid and another invalid, but that the reported metric must correspond to the intended use (Tjepkema-Cloostermans et al., 2025).

Representative examples illustrate this distinction. Jeon et al. evaluated centrotemporal spike-wave detection using epoch-level predictions and a separate within-center recording-level cohort (Jeon et al., 2022). Zhang et al. classified overlapping 1-s segments from 44 h of pediatric EEG and reported an overall accuracy of 87.0%, not greater than 99% (Zhang et al., 2023). Munia et al. evaluated channel- and epoch-based 1-s classifications on the Temple University EEG Events corpus and emphasized F1 score because of class imbalance (Munia et al., 2023). These designs can support screening or candidate generation, but they do not by themselves establish millisecond-level event localization.

Window-level and recording-level models can be clinically useful when their role is clearly defined. For example, a recording-level system may rapidly identify EEGs that require expert review, even if it does not localize every spike. Conversely, an event detector may be valuable for quantifying IED burden but still be insufficient for diagnosis if it performs poorly on normal variants or artifacts. Comparisons should therefore be made within the same task and under similar class balance, recording conditions, and validation design.

Event-level evaluation requires a prespecified matching protocol. The target may be an onset, peak, interval, or multichannel field, and the acceptable temporal tolerance or overlap criterion should be justified. A prediction 50 m from an expert marker is correct only if it satisfies the stated rule. Under one-to-one matching, a single predicted window spanning two adjacent expert-annotated IEDs can match at most one event; the other remains a false negative, and unmatched predictions are false positives. Spatial agreement—such as concordant channel involvement or field distribution—should be reported separately when localization is an intended claim. No universal tolerance has yet been established across all EEG settings.

High epoch-level accuracy should therefore be described as evidence for the corresponding classification task, not as direct evidence of precise IED localization or clinical utility. A more constructive reporting standard is to require authors to identify the unit of analysis, class distribution, matching rule, recording duration, and intended use, and then select metrics that answer that question.

Accuracy, AUC, F1 score, event sensitivity, expert concordance, and FP/h are not interchangeable. Their values depend on prevalence, annotation unit, threshold, matching rule, and test design. Cross-subject validation uses patients not included in training but may still occur within the same institution and acquisition pipeline. External validation instead evaluates an independent institution or dataset, preferably with different hardware, protocols, and patient characteristics. Table 1 therefore summarizes study design and reporting rather than ranking methods by a single numerical score.

**TABLE 1:** Representative studies illustrating differences in task definition, dataset scale, validation design, and outcome reporting.

References/year	Target and modality	Dataset/centers	Recording duration	Annotated IEDs	Validation/evaluation unit	Reported metrics and results	Interpretation/workflow
Primarily internal or within-source validation							
Jeon et al. (2022)	CTSW epoch detection and EEG-level classification; scalp EEG CNN	70 SLECTS patients +70 controls; single center	Each EEG ≥30 min; exact total NR	1,672 annotated CTSWs in the 20-patient event subset	Patient-disjoint within-center development; separate within-center EEG-level cohort	Sensitivity 99.8%; specificity 98.4%; accuracy 99.1%; FDR 0.19/h	Strong performance for CTSW screening in the studied syndrome; not external validation or universal event localization
Zhang et al. (2023)	Four-class 1-s segment classification; pediatric scalp EEG CNN	11 patients; single center	44 h	1,206 IEDs	Random 10-fold cross-validation of overlapping 1-s segments	Accuracy 87.0%; precision 87.6%; recall 86.2%	Segment-level classification; overlapping windows and within-source validation limit external claims
Munia et al. (2023)	Channel- and epoch-based 1-s classification; multi-head CNN	TUEV corpus; 390 patients	Continuous recording hours NR	25,106 channel IED windows; 5,825 IED epochs	Five-fold cross-validation	Channel-based binary F1 87.18%; epoch-based binary F1 73.69%	Useful comparison of analysis units; no independent external test reported
Independent, cross-dataset, prospective, or workflow-oriented evaluation							
Quon et al. (2022)	Intracranial 2-s epoch classification; template matching + CNN	307 subjects in development data; external annotated test set	NR; 2,490 labeled 2-s epochs in development data	1,062 IED-positive epochs	Internal evaluation plus independent external test; epoch-level	Internal F1 0.95; AUC 0.98; external F1 0.71	External decline demonstrates morphology-dependent transfer limits; not continuous-recording FP/h
da Silva Lourenço et al. (2023)	Recording-level ambulatory EEG review from flagged 2-s epochs	100 EEGs; single-center independent test set	Mean 20.6 h/EEG (≈2,000 h total)	NR in test set	Independent from training data; recording-level expert review	97% agreement with original reports; 39/42 IED-positive EEGs identified; 7.1 candidates/h; 2.4 min mean review time	Demonstrates workflow and time-saving value; single reader and single center
Geng et al. (2021)	Intracranial spike classification; LSTM/GRU + AC-GAN	Two real-world iEEG datasets from two institutions	NR	NR in this review	Cross-validation plus cross-dataset testing	High sensitivity and specificity reported; improved with synthetic augmentation	Evidence of cross-dataset transfer; FP/h and prospective workflow outcomes not reported
Lin et al. (2025a)	Multimodal video-EEG IED detection	Prospective test: 149 patients, 3 centers	377 h	9,232 IEDs	Prospective multicenter test; 4-s epochs	AUPRC 0.76–0.78; AUC 0.96–0.98; 0.16–0.31 FP/min at 80% sensitivity; 24% FP reduction at 95% sensitivity	Video reduced artifact-related false positives; synchronized video may limit portability

Abbreviations: AUC, area under the receiver operating characteristic curve; AUPRC, area under the precision–recall curve; FDR, false detection rate; FP, false positive; IED, interictal epileptiform discharge; NR, not reported. Values should not be compared directly across rows because the target tasks, sampling strategies, class distributions, matching rules, and validation designs differ.

### 5.3 Long-term EEG and temporal modeling: Reducing false positives and enhancing clinical applicability

Long-term recordings provide a particularly important test of clinical workflow. Da Silva Lourenço et al. applied a previously trained network to an independent set of 100 ambulatory EEGs with a mean duration of 20.6 h. An expert reviewed only flagged 2-s epochs and reached the same recording-level conclusion as the original report in 97% of recordings; 39 of 42 IED-containing EEGs were identified. The average review time was 2.4 min per recording, with approximately 7.1 candidate detections per hour, corresponding to a reported 50–75-fold reduction in review time (da Silva Lourenço et al., 2023). These results address recording-level triage and time saving rather than complete event-level concordance.

Adaptive template-based systems and deep models can also incorporate limited expert feedback to refine candidate selection (Lodder and van Putten, 2014). Geng et al. evaluated an LSTM-based detector with generative augmentation on two real-world intracranial EEG datasets and included cross-dataset testing (Geng et al., 2021). This provides evidence of transfer between the studied datasets, but synthetic augmentation and cross-institutional testing do not eliminate the need to report event counts, recording duration, false-positive burden, and performance on prospectively collected data.

Lin et al. developed a multimodal model specifically for IED detection—not seizure detection—using synchronized video to help reject movement-related artifacts (Lin et al., 2025a). The prospective test set included 149 patients, 377 h of video-EEG, and 9,232 IEDs from three centers. Across centers, AUPRC ranged from 0.76 to 0.78 and AUC from 0.96 to 0.98; at 80% sensitivity, false-positive rates were 0.16–0.31 per minute. Video features improved precision by 5%–9% and eliminated 24% of false positives at 95% sensitivity. The study therefore provides evidence for multimodal IED detection, although synchronized video may limit portability to settings in which video is unavailable.

These studies also illustrate why workflow and dataset details matter. The ambulatory study used a single expert and a single-center test cohort, so reproducibility across readers and institutions remains uncertain (da Silva Lourenço et al., 2023). Generative augmentation can improve training efficiency, but synthetic waveforms may not represent the full distribution of rare or atypical IEDs (Geng et al., 2021). The prospective multimodal study demonstrated multicenter robustness, yet its false-positive rate is expressed per minute and should be interpreted in relation to recording duration and review strategy (Lin et al., 2025a). None of these limitations negates the contribution of the studies; they delimit the clinical claims that can be made.

### 5.4 From IED detection to classification and spatial localization

Deep learning has also been extended from candidate detection to recording interpretation and spatial classification. SCORE-AI, for example, classifies routine EEGs as normal or abnormal and assigns clinically relevant categories (Tveit et al., 2023). This recording-level task can support triage and standardized reporting, but it should not be conflated with marking every IED.

CNN-based analysis of 1.5-s EEG segments has been used to classify IEDs by cortical region in focal epilepsy. Reported accuracies differed across frontal, temporal, and occipital classes, and multiclass performance was lower than some binary results (Chung et al., 2023). Because the unit of analysis was a short segment, these values describe regional classification of selected samples rather than continuous-recording event localization.

Such models may provide useful spatial information for review and hypothesis generation. Their role in presurgical assessment, however, requires evaluation against clinically accepted localization references and should include the consequences of regional misclassification.

Lower performance for frontal discharges may reflect greater morphological and field variability, but this explanation remains inferential. Generalization should be tested in pediatric, intensive-care, long-term, and different-hardware datasets rather than assumed from routine EEG results. Spatial classification should also be distinguished from source localization, which requires additional modeling and validation.

### 5.5 Cross-modal extensions

Cross-modal work has extended automated analysis to magnetoencephalography (MEG). A supervised model combining temporal and sensor-topographic information has been used to detect focal epileptiform discharges (Fernández-Martín et al., 2025). Such results show technical feasibility, but the task, event-matching rule, and validation population must be considered before comparison with scalp-EEG detectors.

A separate MEG platform was developed to detect, localize, and visualize high-frequency oscillations (HFOs) that may co-occur with IEDs (Cui et al., 2022). An increase in detected HFO events or spatial overlap is not equivalent to improved IED detection, and HFO-specific false positives should be assessed independently.

MEG offers high spatiotemporal resolution but is expensive, infrastructure-intensive, and concentrated in specialized centers. Accordingly, automated MEG analysis is most plausibly positioned as an adjunct for complex presurgical evaluation rather than a general replacement for EEG. No universal numerical threshold of accuracy is sufficient for clinical adoption; performance requirements depend on the intended use, risk, and human oversight.

### 5.6 Wearable and reduced-channel EEG systems

Reduced-channel and wearable EEG systems should be evaluated according to specific use cases. First, prolonged home recording may increase the chance of capturing IEDs for screening or follow-up. Second, repeated recordings may support longitudinal estimation of IED burden in patients with an established diagnosis. Third, wearable systems may triage patients for confirmatory full-montage EEG (Alkofer et al., 2025). Their principal limitation is loss of spatial sampling, phase-reversal information, and source-localization capability; they are therefore generally unsuitable as stand-alone tools for presurgical localization. Sensor noise, intersensor spacing, electrode stability, adherence, and artifact burden must also be considered (Dan et al., 2023). To summarize the technological developments discussed in the preceding sections, Table 2 compares the main automated IED analysis approaches in terms of their core principles, suitable roles, major limitations, and evaluation priorities.

**TABLE 2:** Functional comparison of automated IED analysis approaches and their evaluation priorities.

Approach	Core principle	Suitable role	Main limitations	Evaluation priorities
Rule-based methods	Templates, thresholds, and morphological criteria	Transparent candidate generation and quality control	Sensitivity to artifacts and atypical morphology; limited transfer	Task-specific sensitivity and precision; false-positive burden by setting; external testing
Traditional machine learning	Engineered temporal, spectral, or network features with classifiers	Efficient triage and interpretable feature-based models	Feature dependence and dataset shift	Patient-disjoint splits; cross-center/device testing; candidate burden and review time
Deep learning	Learned representations from raw or minimally processed EEG	Candidate detection, segment classification, and recording triage	Large labeled datasets; domain shift; explanation uncertainty	Define analysis unit and matching rule; calibration; external validation; workflow outcomes
Multimodal artificial intelligence	Integration of EEG with video, MEG, or imaging	Artifact rejection and contextual decision support	Synchronization, infrastructure, and portability	Incremental value over EEG alone; missing-modality robustness; setting-specific false positives
Wearable or reduced-channel EEG	Prolonged monitoring with fewer electrodes	Home screening, longitudinal burden estimation, and triage	Signal quality and reduced spatial information	Diagnostic yield, adherence, artifacts, battery life, and confirmation with full-montage EEG

The appropriate method depends on the intended use. No approach should be considered clinically superior without task-matched evaluation, independent validation, and assessment of the associated review burden.

## 6 Evaluation metrics and dataset optimization for automated IED detection

Overall accuracy and AUC summarize discrimination but do not fully characterize clinical utility, particularly when IEDs are rare relative to background activity (Dessevres et al., 2025; Halford et al., 2018). Appropriate reporting should combine event sensitivity with precision or positive predictive value, F1 score where relevant, calibration, and a measure of false-positive burden. FP/h is important for continuous recordings, but it should not be treated as the only clinically meaningful metric.

The operating threshold and false-positive measure should be matched to the workflow. For a 20-min routine EEG, the number of false candidates per recording and the additional review time may be most intuitive. For 24-h ambulatory EEG, FP/h, candidate clustering, and total review time become more informative. In intensive-care monitoring, false alarms per patient-day, alarm duration, and alarm fatigue may be relevant alongside sensitivity. Presurgical review may prioritize sensitivity and spatial concordance. A single universal acceptable FP/h is therefore unlikely to apply across settings.

Event-level assessment should define the annotated object and the matching rule, whereas recording-level assessment should state how candidate events are aggregated into a final EEG classification (da Silva Lourenço et al., 2021). Dataset descriptions should include the number of patients, total recording hours, annotated IED count, prevalence, montage, acquisition system, and clinical setting. Patient-disjoint validation within one center assesses cross-subject performance but does not constitute external validation. Independent testing across institutions, hardware, and acquisition protocols provides stronger evidence of generalizability (Falach et al., 2024; Mansilla et al., 2024). Multicenter datasets with multi-expert event annotations are especially valuable, but the degree of independence between training and test centers must be reported explicitly (Lin et al., 2025b).

### 6.1 Methodological pitfalls and limitations

Several recurrent methodological limitations affect the literature. Small or demographically narrow datasets increase the risk of domain bias. Annotation disagreement arises from multiple sources, including montage, filter settings, artifacts, reader experience, access to clinical information, and differences in morphological criteria. Biological heterogeneity may also contribute, but current evidence does not justify attributing disagreement primarily to glial state or receptor balance. Studies should report the annotation protocol, number and expertise of readers, adjudication procedure, and inter-rater statistics. They should also separate training, validation, and test data at the patient level and prevent overlap introduced by segmented windows. Finally, external validation should be clearly distinguished from internal cross-validation.

### 6.2 Explainability and regulatory considerations

Saliency maps, gradient-based visualizations, attention weights, and feature-attribution methods can show which input regions influenced a model, but visualization is not equivalent to interpretability. These outputs may be unstable across random seeds, minor signal perturbations, or correlated features, and attention weights do not necessarily provide a causal explanation. Evaluation should test reproducibility, sensitivity to perturbation, correspondence with accepted IED morphology and field distribution, and usefulness to clinicians. Inherently interpretable or example-based models may complement *post hoc* visualization, but their explanations also require validation.

Clinical deployment also requires compliance with the applicable medical-device framework, transparent documentation of the intended use, and evidence that performance is reproducible in the target population and hardware environment. Cybersecurity, software version control, model updating, failure-mode analysis, and clear allocation of clinical responsibility are relevant. Regulatory requirements vary by jurisdiction and device claim; therefore, no single validation design can automatically guarantee regulatory clearance.

### 6.3 Benchmarking commercial systems and real-world clinical workflows

Commercial platforms such as Persyst, Encevis, and BESA are already used as assistive tools in some EEG workflows. Head-to-head evaluation has shown differences in sensitivity, specificity, and agreement with reference interpretation (Reus et al., 2022). Such comparisons are informative only when software versions, thresholds, recording types, and reference labels are specified. Commercial systems may combine heuristic features, clustering, and machine-learning components; their performance should be reported empirically rather than characterized as uniformly outdated or state of the art.

Automated quantification may also support longitudinal assessment. Changes in IED burden around seizures or during tapering of antiseizure medications (ASMs) have been investigated as dynamic markers of seizure risk (De Stefano et al., 2022). In a series of 267 long-term EEG recordings, Encevis showed high sensitivity for ictal and interictal activity but limited specificity as a stand-alone system, reinforcing the need for expert review (Tsereteli et al., 2025). These findings support decision assistance, not autonomous treatment decisions.

Human-in-the-loop evaluation should measure more than AUC. Relevant outcomes include candidate events presented per hour or per recording, the proportion accepted, rejected, or modified by clinicians, review time, missed clinically important events, inter-rater agreement, and the proportion of final reports or management decisions changed after AI assistance. Semi-automated workflows can reduce review burden while retaining expert adjudication (Kural et al., 2022), but their benefit should be established prospectively and compared with usual care.

## 7 Future perspectives

Automated IED analysis has progressed across routine EEG, ambulatory EEG, intracranial EEG, video-EEG, and MEG, but real-world adoption remains constrained by heterogeneous datasets, annotations, tasks, and metrics (Tjepkema-Cloostermans et al., 2025; Zhang et al., 2023; Geng et al., 2021; Fernández-Martín et al., 2025). Biological mechanisms remain relevant as a source of hypotheses. In animal models, complement-associated microglial synapse elimination has been linked to synaptic dysfunction and epileptiform activity, and C1q-targeted interventions have modified selected electrophysiological outcomes (Jeong et al., 2025; Holden et al., 2021). These findings do not establish that a scalp-EEG model can measure synaptic pruning in an individual patient. Automated morphology and burden estimates should therefore be treated as electrophysiological phenotypes that may be correlated with biological measurements in future studies, not as direct readouts of synaptic integrity.

The field is moving from proof-of-concept detector development toward data quality, generalization, and workflow integration. Architecture innovation remains important, but it cannot compensate for nonindependent data splits, incomplete reporting, or a test population that does not represent the intended use. False-positive output may contribute to overinterpretation and unnecessary treatment, whereas missed abnormalities may delay further evaluation (Kara et al., 2020). Clinical value should therefore be established through task-matched, prospectively defined outcomes.

Future benchmarks should distinguish window classification, event localization, recording classification, and source localization. For event detection, the annotation target, temporal tolerance or overlap rule, one-to-one matching procedure, and spatial criterion should be prespecified. For generalization, patient-disjoint internal testing should be reported separately from external testing at independent institutions. Data repositories should document acquisition hardware, montage, filters, age, syndrome, vigilance state, medications, and artifact burden, and should use strict partitioning to avoid leakage (Tatum, 2013; Fürbass et al., 2020).

Statistical evaluation should be customized to the clinical workflow. Alongside discrimination metrics, studies should report calibration, candidate burden, review time, clinician acceptance and rejection rates, and diagnostic or management impact. Multi-expert consensus and inter-rater measures are important, but model performance should not be treated as exceeding an ill-defined human gold standard when experts themselves disagree. Common external test sets can facilitate comparison, provided that they do not become repeatedly optimized *de facto* training sets (Fürbass et al., 2020).

**FIGURE 4:**
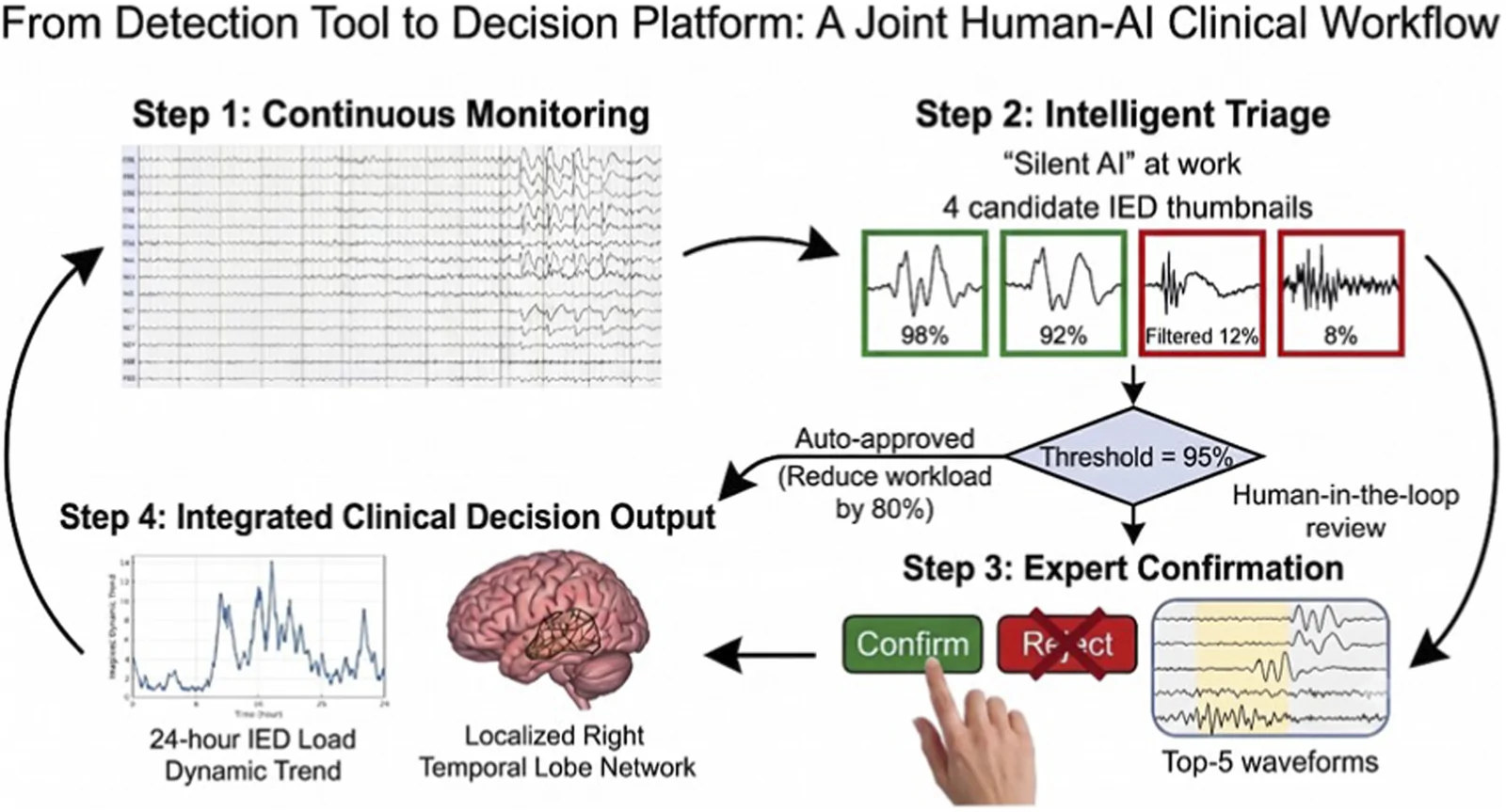
Proposed human–AI workflow for automated IED review.

As illustrated in Figure 4, continuous EEG is processed by an assistive triage system that ranks candidate IEDs and communicates model confidence or uncertainty. Thresholds may be configured for the intended workflow, but high-confidence events are not automatically converted into clinical findings. Neurophysiologists review, confirm, reject, or modify candidates and remain responsible for the final interpretation. Validated events can then be summarized as IED burden, temporal trends, and spatial distributions. Evaluation of this workflow should include candidates per hour, clinician acceptance and rejection rates, review-time reduction, missed events, and effects on the final report.

Human–AI collaboration should be designed as decision support rather than unqualified automation. Candidate ranking and uncertainty display may help readers navigate long recordings, but trust requires evidence of robustness across hardware, populations, and artifacts. Interfaces should preserve access to the surrounding EEG, montage changes, filters, and clinical context. The clinician should be able to reject or modify every output, and system performance should be monitored after deployment (Abdi-Sargezeh et al., 2021).

A practical macro-to-molecular research framework would require prospective, spatially matched data rather than inference from EEG alone. In surgical cohorts, preoperative scalp EEG and intracranial EEG or stereoelectroencephalography could be aligned with electrode coordinates and the location of resected tissue. Single-cell or single-nucleus RNA sequencing and spatial transcriptomics could then characterize cell types and pathways within sampled regions (Liu et al., 2024; Chen et al., 2026). Analyses could test whether local IED burden, morphology, propagation, or sleep-state dependence correlates with independently measured molecular features. Such studies must account for tissue sampling, antiseizure medications, anesthesia, pathology, and the imperfect correspondence between electrode fields and resection specimens. Until replicated in prospective multicenter cohorts, genotype, ion-channel expression, or inflammatory state should not be inferred from automated IED output in clinical practice.

## 8 Conclusion

IEDs are established electrophysiological biomarkers, and automated analysis may reduce the burden of reviewing prolonged, multichannel EEG recordings. The field has progressed from template-based and handcrafted-feature methods to deep learning and multimodal systems; however, these approaches address different tasks and cannot be ranked using a single performance metric. Window-level accuracy, event-level sensitivity and F1 score, recording-level agreement, false positives per hour, and review-time reduction answer distinct clinical questions, while cross-subject testing within one center represents internal rather than external validation. Further translation requires standardized reporting of the unit of analysis, temporal and spatial event-matching rules, recording duration, annotated IED count, class balance, operating threshold, and clinically appropriate false-positive burden; diverse multicenter benchmarks with multi-expert annotations and independent testing across institutions, hardware, and acquisition protocols; and prospective evaluation of human–AI workflows using candidate burden, clinician acceptance or rejection, review time, missed clinically important events, final-report agreement, and downstream clinical impact. Biological mechanisms, including neuroinflammation, complement-associated synaptic remodeling, chloride homeostasis, and genetic variation, can inform hypothesis generation and cohort stratification, but current evidence does not justify interpreting AI-derived IED phenotypes as direct readouts of molecular states. With rigorous task definitions, transparent reporting, independent validation, and continued expert oversight, automated IED analysis may evolve from candidate detection toward reliable decision support for EEG interpretation, longitudinal monitoring, and mechanistic research on epileptic networks.
